# Minimization of Biosynthetic Costs in Adaptive Gene Expression Responses of Yeast to Environmental Changes

**DOI:** 10.1371/journal.pcbi.1000674

**Published:** 2010-02-12

**Authors:** Ester Vilaprinyo, Rui Alves, Albert Sorribas

**Affiliations:** 1 Fundació Dr Ferràn-Hospital de Tortosa Verge de la Cinta, Tortosa, Spain; 2 Departament de Ciències Mèdiques Bàsiques, Institut de Recerca Biomèdica de Lleida (IRBLLEIDA), Universitat de Lleida, Lleida, Spain; University of Illinois at Urbana-Champaign, United States of America

## Abstract

Yeast successfully adapts to an environmental stress by altering physiology and fine-tuning metabolism. This fine-tuning is achieved through regulation of both gene expression and protein activity, and it is shaped by various physiological requirements. Such requirements impose a sustained evolutionary pressure that ultimately selects a specific gene expression profile, generating a suitable adaptive response to each environmental change. Although some of the requirements are stress specific, it is likely that others are common to various situations. We hypothesize that an evolutionary pressure for minimizing biosynthetic costs might have left signatures in the physicochemical properties of proteins whose gene expression is fine-tuned during adaptive responses. To test this hypothesis we analyze existing yeast transcriptomic data for such responses and investigate how several properties of proteins correlate to changes in gene expression. Our results reveal signatures that are consistent with a selective pressure for economy in protein synthesis during adaptive response of yeast to various types of stress. These signatures differentiate two groups of adaptive responses with respect to how cells manage expenditure in protein biosynthesis. In one group, significant trends towards downregulation of large proteins and upregulation of small ones are observed. In the other group we find no such trends. These results are consistent with resource limitation being important in the evolution of the first group of stress responses.

## Author Summary

Although different environmental stresses trigger specific sets of protective changes in the gene expression of yeast, the adaptive responses to these stresses also share some common features. We hypothesize that minimization of metabolic costs may contribute to shaping such adaptive responses. If this is so, then such pressure should be more noticeable in the costliest biosynthetic processes. One of these is protein synthesis. Thus, we analyze the set of genes and proteins whose expression changes during the responses and look for evidence to support or falsify our hypothesis. We find that protein properties that are indicative of protein cost correlate to changes in gene expression in a way that is consistent with that hypothesis for a large number of adaptive responses. However, if changes in gene expression are small during the adaptive response, we find no evidence of protein cost as a factor in shaping the adaptive response.

## Introduction

Unicellular organisms are sensitive to environmental challenges. Their internal milieu acts as a buffer against such changes by mounting an adaptive response involving modifications at different cellular levels. Appropriate adaptive responses require intracellular signaling, changes in the conformation and activity of proteins, changes in transcription and translation of genes, etc. [1]. Many of the cellular modifications that characterize any adaptive response are due to the need for acquiring new protein functionalities while shutting down other protein functionalities that are not required in the new conditions. These changes ultimately fine tune the mechanisms and processes that allow the cell to function appropriately and survive under changing environments.

Such fine tuning is shaped by various functional requirements and physiological constraints. The functional requirements are a result of the specific demands that are imposed on cell survival by the environment. On the other hand, the physiological constraints are defined by the limits within which the cell is physically capable of changing the activity of its component parts to meet the functional requirements. From a global point of view, adaptive responses can be seen as a multi-optimization problem because cells evolved appropriate responses to cope with different types of stress, while optimizing different parts of its metabolism for each of those responses [2],[3]. For example, cells simultaneously have to increase the concentration of specific metabolites and proteins, while decreasing the concentration of other components to prevent an increase in the concentration of unneeded metabolites. Such an increase could strain cell solubility capacity or increase spurious reactivity to dangerous levels. These and other functional constraints are likely to provide sustained evolutionary pressures that ultimately select a specific gene expression profile that leads to suitable adaptive responses.

With these arguments in mind, it is thus important to identify the functional requirements and quantitative physiological constraints that may significantly shape adaptive responses. Among others, minimization of energetic expenditure plays an important role in cells growing exponentially in a rich medium. Several signatures that are consistent with minimization of metabolic cost have already been identified in the properties of the set of proteins that is expressed when cells are growing in rich media (*basal conditions*).

For example, genes coding for proteins that are highly abundant under basal conditions have a pattern of synonymous codon usage that is well adapted to the relative abundance of synonymous tRNAs in the yeast *S. cerevisiae* and in *Escherichia coli* [4],[5].

Another signature that is found in genes that are highly expressed under basal conditions is a sequence bias that minimizes transcriptional and translational costs [6]. This minimization of metabolic cost is further observed in the relative amino acid composition of abundant proteins under the same conditions. These proteins are enriched with metabolically cheaper amino acids [7].

A final example of a general signature is the codon bias of long genes. This bias is such that the probability of missense errors is reduced during translation [6],[8],[9]. These biases suggest that reducing overall costs in metabolism, whenever possible, may significantly increase cellular fitness. This view is consistent with the observation that small changes in gene expression affecting the levels of protein synthesis influence the fitness of specific *E. coli* strains [10].

This body of results strongly supports the notion that metabolic cost acts as a selective pressure in shaping the properties of cells growing in a rich medium, in absence of environmental stresses. Thus, one might ask if minimization of metabolic cost is also an important factor in the evolution of adaptive responses to stress conditions. It is predictable that this evolutionary pressure might leave stronger signatures in adaptive responses that require the use of higher ATP amounts by the cell, such as adaptation to heat, weak organic acids, or NaCl. In these three cases, it has been reported that ATP concentrations decrease due to a high energy demand [11].

Given that protein synthesis is one of the costliest biosynthetic efforts for the cell [12], the minimization of metabolic cost might have biased the properties of proteins whose expression change during adaptation. Therefore, here we ask the following questions. Is there a signature that is consistent with a selective pressure for minimizing metabolic cost in proteins synthesis during adaptive responses to stress? Can one find general signatures in the physicochemical properties proteins and in the expression patterns of genes that are involved in the adaptive response to different environmental challenges? If so, what physiological constraints are consistent with those signatures?

We address these questions by investigating how is the value of several properties of proteins (size and molecular weight of proteins, codon adaptation index, aromaticity, average cost per amino acid, etc.) related to changes in gene expression levels during various environmental changes.

We find that genes whose expression is upregulated during different types of adaptive responses tend to code for proteins that are small, while genes whose expression is downregulated during the same responses tend to code for proteins that are large. This is a signature that is consistent with a selective pressure for minimizing metabolic cost in proteins synthesis. It is more significant in adaptive responses where changes in gene expression levels affect a large fraction of the genome. To our knowledge, this is the first general and global signature that has been identified for the properties of proteins involved in adaptive responses to stress.

## Materials and Methods

### Databases of gene expression, protein abundance and protein properties

#### Microarray data

Data from 249 published microarray experiments that measure changes in yeast gene expression under a battery of different environmental stresses [13],[14] have been analyzed. Changes in gene expression during responses to the following environmental signals are considered: heat shock, menadione, hydrogen peroxide, DTT, diamide, acid and alkali stresses, changes of carbon sources (C Source), NaCl, diauxic shift, nitrogen (↓N) and amino acid depletion (↓AA), high sorbitol concentration (Sorbitol) and return to normal osmolarity after being subject to high sorbitol concentrations (hereafter referred to as ↓Sorbitol), and stationary phase at 25° and 30°C (ST25 and ST30 respectively). Details on the experimental conditions can be found in the literature [13],[14] and the data itself is publicly available.

#### Functional classification of genes

Categorization of protein function, biological process, and location was done using Gene Ontology (GO) terms provided by the Saccharomyces Genome Database (SGD, http://www.yeastgenome.org) tool Go Ontology Slim Mapper. Higher level (Broad classification) GO terms were identified using Super Go-Slim. The ORFs were further classified using the Yeast Go-Slim classification. This classification includes details about the major biological processes, functions, and cellular components of *S. cerevisiae*. We do not examine individual pathways, because in most cases such pathways have such a small number of proteins that the statistical significance of the changes cannot be assessed.

#### Protein properties, mRNA length, abundance and half-life data

Whole-genome data for basal protein abundance [15], basal protein half-life [16], and mRNA were obtained from the literature (mRNA[A] from [17] and mRNA[H] from [18]). In all cases, the data pertain to yeast growing exponentially in a rich medium (basal conditions).

#### Other protein properties

The physicochemical properties of proteins as well as the list of protein complexes in yeast were obtained from the SGD ftp site [19]. Different properties of proteins that we analyze are: length, molecular weight, isoelectric point, Codon Adaptation Index (CAI) [20],[21], Codon Bias Index (CB) [22], and Frequency of Optimal Codons (FOP) [23], protein hydropathicity (as measured by the GRAVY score) [24], aromaticity score [25], and average amino acid cost of proteins [7].

#### Selecting the set of proteins to analyze

According to the available data [15], under optimal growth conditions the abundance of a protein ranges from fewer than 50 molecules to more than 10^6^ molecules per cell. The experimental methods for determining protein amount have limited detection sensitivity at low abundance. Because of this, determinations for proteins whose abundance lies on the lower boundaries of detection will most likely have the largest relative errors. To avoid that this error influences the analysis we ranked all proteins by abundance. Then we selected the proteins to include in our study, starting at the most abundant in the list and moving down to less abundant proteins. When 99.5% of all protein mass in the cell is included in the list of proteins to analyze, we discard the remaining proteins.

### Estimating maximum level of gene expression change from microarray experiments

The microarray data we analyze provide information regarding relative up and downregulation (UpCF and DownCF, respectively) of gene expression with respect to a pre-stress control condition. To facilitate comparison between upregulated and downregulated genes, we use the inverse of the ratio for downregulated genes. Thus, all values for the ratios of changes in gene expression discussed below are greater than 1.

Changes in gene expression during stress responses are dynamic and, for the most part, transient. Because of this, we take the maximum value of up or downregulation as an approximated measure of the maximal change in gene expression during the transient stress response.

Changes in gene expression are underestimated for genes that undergo very strong up or downregulation, due to intrinsic limitations of the microarray technology [26]. To minimize any errors that may come from this limitation we use the 98^th^ quantile of all the ratio values for a given gene as a proxy of its maximum UpCF or DownCF.

### Analysis of gene expression changes

#### Statistical comparison of gene expression changes

Spearman rank correlations are used to characterize the dependencies between properties of proteins and changes in gene expression to a first approximation. However, this statistical index has some constraints that limit its usefulness for our analysis. First, the high number of observations may lead to statistically significant results even with low correlation values. Second, it is very sensitive to noisy data. Third, distributions that are asymmetric and have heavy long tails, such as those of our datasets, may influence the correlations and produce false results. All these constraints may lead to erroneous interpretation of the results. Thus, although correlation analysis gives a global description of the possible trends, such an analysis needs to be complemented with more detailed methods in order to support an interpretation of the set of results.

Thus, to further assess the biological relevance of the correlations we use the following procedure. First, and because the distribution of each of the considered properties has long tails, we select the values that fall within the 80% interquantile for the property of interest. Then, we divide this range into 3 groups. Finally, we compute the ranks of the change in gene expression between the two extreme groups obtained by this criteria, discarding the middle group, and test for distribution differences by using the Mann-Whitney U rank-sum test. In the set of UpCF proteins, a positive z for this test means that the group with high values for the property is less upregulated than the group with low values for the property. In the set of DownCF proteins, a positive z for this test means that the group with high values for the property is more downregulated than the group with low values for the property (see for instance Tables 1–2).

**Table 1:** Comparison of changes in gene expression between low and high abundant proteins, for each of the environmental conditions.

Environmental condition	Up- CF			Down- CF			Thresholds	
	z		P	z		p	Lower	Upper
ST25	+	4.19	***	+	21.54	***	7.79	14.91
ST30	+	7.63	***	+	19.20	***	6.17	11.70
Heat	+	8.69	***	+	10.54	***	7.27	13.89
↓N	+	3.88	***	+	18.95	***	6.90	13.12
Peroxide	+	4.19	***	+	13.35	***	6.17	11.70
NaCl	+	5.23	***	+	6.49	***	7.04	13.39
Diauxic	+	1.82	***	+	16.70	***	6.96	13.26
↓AA	+	1.04	0.15	+	14.88	***	6.13	11.60
Sorbitol	+	4.04	***	+	15.91	***	7.27	13.89
Alkali	+	1.74	***	+	6.54	***	5.96	11.22
DTT	−	11.66	***	+	6.53	***	6.98	13.31
Diamide	−	2.77	***	+	11.66	***	8.63	16.65
Menadione	−	4.14	***	+	3.47	***	7.56	14.49
Acid	+	6.29	***	+	6.36	***	7.37	14.07
C Source	−	12.66	***	+	6.09	***	7.03	13.40
↓Sorbitol	−	7.87	***	−	6.53	***	5.50	10.32

We identify the extreme group values for abundance and use the Mann-Whitney analysis for characterizing positive or negative associations with gene expression levels, as detailed in the methods section. Lower and Upper concentration thresholds indicates the cutoff limits for selecting low abundance proteins and high abundance proteins. A positive z indicates that proteins in the Lower group present higher up-expression and lower down-expression than those in the Upper group as compared by the Mann-Whitney analysis. A negative z indicates the opposite result. The corresponding p-values obtained using this test are indicated as (***) if p<0.05. Abundance is divided by 10^3^ pr/cell.

**Table 2:** Comparison of changes in gene expression between short and large proteins, for each of the environmental conditions.

Environmental condition	Up- CF			Down- CF			Thresholds	
	z		p	z		P	Lower	Upper
ST25	+	5.76	***	+	2.15	***	408	653
ST30	+	4.23	***	−	2.08	***	416	665
Heat	+	1.67	***	+	4.53	***	419	672
↓N	+	9.91	***	+	3.69	***	415	662
Peroxide	+	4.72	***	+	8.11	***	421	677
NaCl	+	3.05	***	+	4.20	***	415	671
Diauxic	+	9.47	***	+	3.62	***	405	639
↓AA	+	1.61	0.05	+	2.65	***	416	667
Sorbitol	+	1.58	0.06	−	1.19	0.12	416	666
Alkali	+	2.89	***	+	2.73	***	434	703
DTT	+	13.69	***	+	5.64	***	411	658
Diamide	+	11.07	***	+	7.15	***	408	660
Menadione	+	7.51	***	−	4.91	***	435	702
Acid	+	1.03	0.15	−	1.57	0.06	431	691
C Source	−	15.20	***	−	8.79	***	413	662
↓Sorbitol	−	7.72	***	−	8.64	***	415	656

We identify the extreme group values for length and use the Mann-Whitney analysis for characterizing positive or negative associations with gene expression levels, as detailed in the methods section. (See legend in Table 1).

#### Descriptive plots

*Moving-quantile plots* are used to smooth the noise and allow for a visual analysis of the global tendencies in the changes of gene expression. We use a window of 300 proteins for the moving-quantiles.

*Quantile-quantile plots* are used to compare the distribution of the values of two samples. To build such plots, one determines the quantiles for each sample separately. Then the values of the variable that correspond to the same quantile in each separate sample are plotted one against each other. A reference line with a slope of 1 helps in checking for differences between distributions. If the two lists have a similar distribution, the points will fall near the reference line.

#### Computational tools

All analyses were done using our own functions implemented in *Mathematica* version 7.0.

## Results

As discussed in the Introduction, previous authors report clear trends between different properties of proteins and their basal abundance in yeast growing exponentially on a rich medium (*basal conditions*) [6],[7],[8],[9]. In this work we evaluate the existence of similar signatures in the proteins that are involved in the adaptive response of yeast to stress. Before presenting the specific results, and due to the complexity of the analysis, it is worth it to briefly outline the strategy we follow and its rationale. There are four main steps:

1. Characterize how the selected protein properties (see Methods) correlate with each other. This is important because it allows us to control later on whether a relationship between changes in gene expression and a given property may be the result of a secondary correlation between properties of proteins or not.
2. Use changes in gene expression as a proxy for changes in protein level and investigate how such changes correlate with the protein properties considered in this work. In order to assure consistency of the results, and because the signal to noise ratio is low for our purposes, a three tiers analysis of the data is required. First, we perform a correlation analysis between changes in gene expression and the different values of the protein properties. However, even a statistically significant correlation coefficient can be misleading because these coefficients are almost always significant in large datasets. Furthermore, a correlation coefficient describes an inhomogeneous set of data with a point measure, which is an important limitation in our case. Second, and to overcome this limitation, we use the Mann-Whitney test to compare the bulk differences in gene expression between proteins that have extreme values for the property of interest. This test enables us to appropriately deal with the asymmetrical and heavy tailed nature of the distributions found in our datasets. Finally, we use moving-quantile plots to represent changes in gene expression as a function of the different properties. This allows us to do both, resolve any apparent contradictions that may arise between the correlation analysis and the Mann-Whitney analysis, and have a finer detail representation of our data.
3. Results from 2 are consistent with economy being an important factor in shaping different stress responses. To further investigate this issue we define a quantitative index that estimates the cost of changing protein expression. We use clustering analysis and discriminate analysis to investigate how the different stress responses behave with respect to this index.
4. The results from the previous steps suggest that there are two types of stress responses in regards to the amount of changes in gene expression observed during the response. Therefore, we pool together the gene expression changes in stress responses of the same type, in order to have a stronger signal. We reanalyze the pooled data in order to ensure consistency between this set of responses and the results for the individual responses. Then, we use the pooled dataset to investigate how molecular complexes and protein function might influence our results by analyze the proteins in different Gene Ontology (GO) categories.

We now discuss the results of the analysis in detail.

### Correlation between different protein properties

Some of the protein properties we consider are strongly correlated (see Table S1). For example, different measures of codon preference towards the major tRNA isoacceptors, such as CAI, CB, and FOP, are highly correlated (*r* = 0.83–0.97). Length and molecular weight of proteins are, in practice, equivalent. Protein and mRNA abundance show a correlation of *r* = 0.56. Protein abundance is also positively correlated to CAI, to CB and to FOP (*r* = 0.53–0.54), and to mRNA abundance under basal growth conditions (*r* = 0.60–0.64). Similarly, average amino acid cost (ACPA) is highly correlated to aromaticity (*r* = 0.84), because the most expensive amino acids are aromatic. Thus, if a protein has a high percentage of aromatic amino acids (which is proportional to aromaticity) it will have a larger average cost per amino acid than proteins with lower percentage of aromatic amino acids.

### Relation between changes in gene expression and protein properties

The only type of data that is available for both, the entire genome and a comprehensive set of yeast adaptive responses, is gene expression data from microarray experiments [13],[27]. Thus, in order to search for trends between the adaptive responses and the protein properties, we analyze how the value of those properties is related to the changes in gene expression during the response. We analyze microarray data for fourteen stress responses and two control conditions (change in carbon source — C Source — and return to basal conditions after osmotic shock — ↓Sorbitol).

#### Correlations between gene expression and protein properties

**Figure 1:**
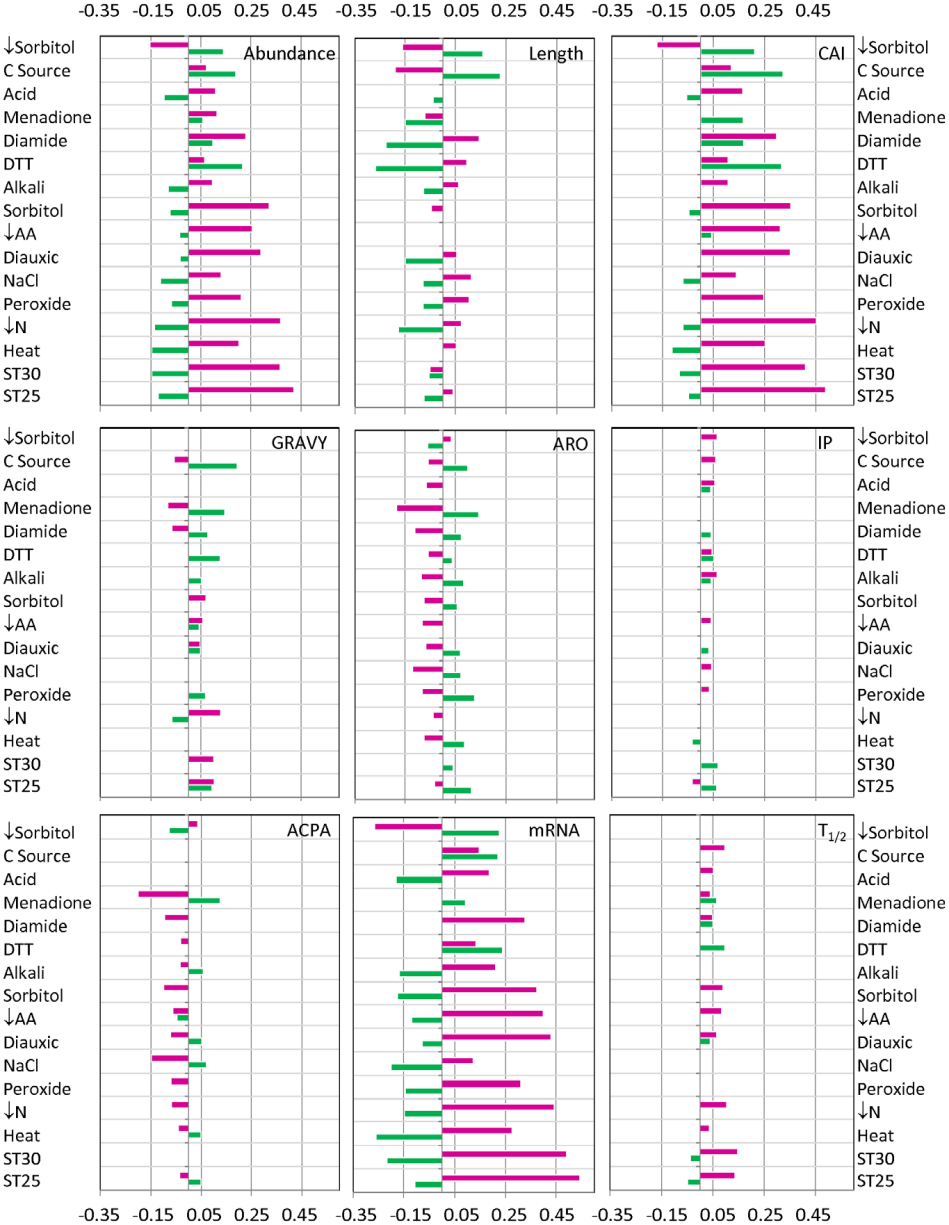
Spearman rank correlation between properties of proteins and changes in gene expression for each stress condition. Only the results with statistical significance (p<0.05) are shown. Green bars correspond to upregulation. Purple bars correspond to downregulation.

As a first step in the analysis of the relationship between changes in gene expression and each of the different protein properties, we evaluate how the maximum change in mRNA level for the microarray data of each stress response correlates to the property of interest. This is done by calculating the Spearman rank correlation coefficient. Upregulated and downregulated genes are analyzed independently. The results are summarized in Figure 1.

Those results reveal substantial diversity in the properties of the proteins that are induced or repressed during the various responses to stress. Despite this, for most adaptive responses we found a similar pattern for the relationship between changes in gene expression and protein abundance, protein length, codon adaptation index, or mRNA abundance. The value for each of these properties tends to decrease if the gene is more upregulated and increase if the gene is more downregulated.

In 11 stress responses, the most upregulated proteins tend to be less abundant under basal conditions. In 15 stress responses, the more downregulated proteins tend to be more abundant under basal conditions. As expected because of the high correlation between protein abundance and CAI or basal mRNA abundance, the correlation between these properties and changes in gene expression is similar to those for abundance. Surprisingly, and although abundance and length are negatively correlated (Table S1) we find that in 11 cases the most upregulated proteins tend to be short and that in 9 cases the most upregulated tend to be large.

Correlations between changes in gene expression and GRAVY, Aromaticity, IP, ACPA or protein half-life are either non-significant or weak.

#### Comparison of expression changes between groups with extreme values for properties

An analysis of the results finds that the properties that are more strongly correlated to changes in gene expression are those that can be considered as a proxy for cost of protein synthesis. Because of this we focus the next step of our analysis on those properties, which are protein length, protein abundance, CAI and T_1/2_. The relationship between each of these properties and cost of protein synthesis can be explained as follows. First, abundant proteins require more resources to synthesize and maintain than proteins that are present in low copy numbers. The same is true for proteins with low T_1/2_. Second, longer proteins are metabolically more expensive to synthesize than shorter proteins because they use more amino acids per peptide chain. Third, the codon adaptation index can also be a proxy for the rate of synthesis of a protein, given that proteins with a high CAI are more likely to be highly expressed than proteins with a low CAI.

If cost of protein synthesis is an issue that influences evolution of stress response, then proteins that are more expensive should be more strongly repressed and proteins that are cheaper should be more strongly upregulated. Therefore, one needs to analyze if changes in gene expression are different between the cheaper and the most expensive proteins. The Mann-Whitney analysis of the extreme groups for each property, although less intuitive than the correlation analysis, allows us to perform such a comparison. Thus, we are analyzing the groups of proteins in which the signal is likely to be strongest.

The results for abundance and length are summarized in Tables 1–2. They confirm that the repression of abundant and long proteins and up-expression for low-abundant and short proteins is observed for most stress responses. Interestingly, the two control conditions (↓Sorbitol and C Source) show correlations that are opposite to those observed for most stress responses. In addition, adaptive responses to stresses by DTT and Diamide (once) and Menadione and Acid (twice) show an absence of correlation between changes in gene expression and protein length/protein abundance.

Results for CAI are almost identical to those for abundance and we do not find any clear trend for T_1/2_ (data not shown).

#### Visualization of trends

**Figure 2:**
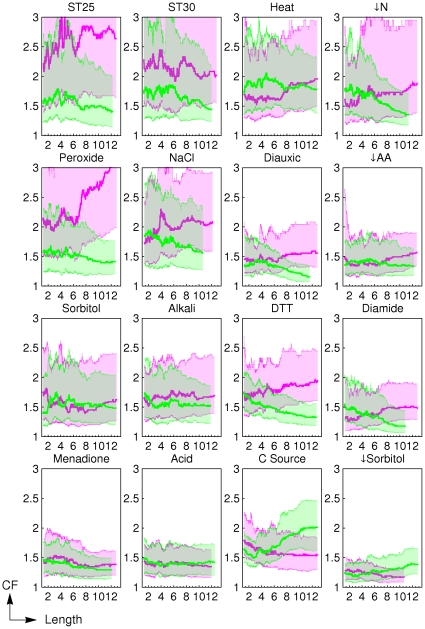
Change-folds of genes with respect to their length. Plots show the moving-median using a window of 300 elements. Colors: Green for upregulation and purple for downregulation. Length unit is 10^2^ amino acids. The lines represent the moving median plots. The shaded areas represent the regions from quantile 0.25 to quantile 0.75. Note that in most cases there is an upper limit to the length of upregulated proteins. This limit is smaller than the limit found for to the length of downregulated proteins.

In order to have a finer detail representation of our data and resolve any apparent contradictions between the correlation analysis and the Mann-Whitney analysis (for example compare the results for heat shock response between Figure 1 and Table 2) we use moving-quantile plots. Changes in gene expression vs. either protein length or abundance are shown in Figures 2 and S1, respectively. This visualization method clearly shows that stress responses with lower correlations in Figure 1 and Tables 1–2 have smaller transcriptional changes. The results from the Mann-Whitney analysis are more consistent with the slopes of moving-quantile plots than the correlation analysis. For example, correlations between upregulation of expression and protein length for ST25, ST30 and heat shock are low or null (Figure 1). However, the Mann-Whitney analysis shows that the changes in gene expression in the group of small proteins are significantly different from those in the group of large proteins. The moving-quantile plots for these conditions show a negative slope for the green lines that is stepper for quantile 0.75 (upper green band in Figure 2).

Those plots also permit identifying responses where downregulation spans a longer range of protein lengths and abundances than upregulation. For example, in Figure 2 for conditions ST25, ST30, ↓N, NaCl, Diauxic, ↓AA, Alkaly, Diamide and Menadione the green line is shorter than the purple line.

By and large, only weak relationships are found between change-fold and T_1/2_ (Figure S2). As expected from their high correlation, CAI and protein abundance correlate to change-fold in a similar way (Figure S3).

### Similarities across stress responses

Short proteins with a high relative composition of metabolically cheaper amino acids are highly abundant under basal conditions, which is consistent with the hypothesis that lowering protein cost is a driving force in shaping the protein complement of yeast in those conditions [7]. It is also well known that the process leading up to protein synthesis is one of the costliest components of cellular metabolism [12] and that during response to many environmental stress signals yeast shuts down gene expression and decreases the number of ribosomes [13],[14].

It has been proposed that gene expression profiles have signatures that are specific to the conditions under which they have evolved [2],[28]. If metabolic cost in general, and cost of protein synthesis in particular, is a significant factor in shaping adaptive profiles, then one might expect that the stronger the resource limitation is, the larger its signature will be. It would then be reasonable to expect that adaptive responses where a resource limitation exists may have similar qualitative bulk expenditure in protein synthesis.

To find support for this hypothesis we must estimate that cost for the different stress responses. Changes in protein levels can be roughly estimated over the whole genome by the changes in the levels of gene expression [29]. Thus, an index 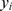 that approximately estimates the changing costs of protein synthesis during a given adaptive response *i* can be defined as:
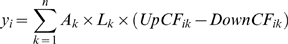
(1)


In this equation *A_k_* is the basal abundance of protein *k* and *L_k_* is the primary sequence length of that protein *UpCF_ik_* and *DownCF_ik_* represent the change-fold of up- or downregulation of the gene that codes for protein *k*.

It is likely that specific functional requirements during any given stress response will lead to the synthesis of new proteins whose functionality is required for survival under the new conditions. By calculating a cost index for each of the twenty five Gene Ontology (GO) categories of cellular components defined in the SGD Slim Mapper Tool, we can analyze if the requirement for new functions is restricted to specific categories of the GO classification or not. Such a discriminating cost index can be defined as:
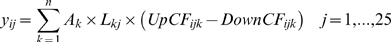
(2)The index 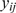 refers to stress condition 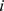 and GO category 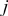. For each protein 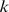 within the GO category 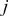, the up- (*UpCF_ijk_*) or down-change fold (*DownCF_ijk_*) is multiplied by its length 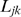. If in the GO category 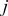 the expression of genes coding for small proteins is preferably upregulated and the expression of genes coding for large proteins is preferably downregulated, the index 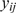 will be negative. This index provides a rough bulk estimate of how much a cell invests in synthesizing new proteins (the upregulation term) subtracting how much the cell saves by decreasing the synthesis of other proteins (the downregulation term). The index under basal conditions is 0 because the difference between up- and down-expression is null in that case.

**Figure 3:**
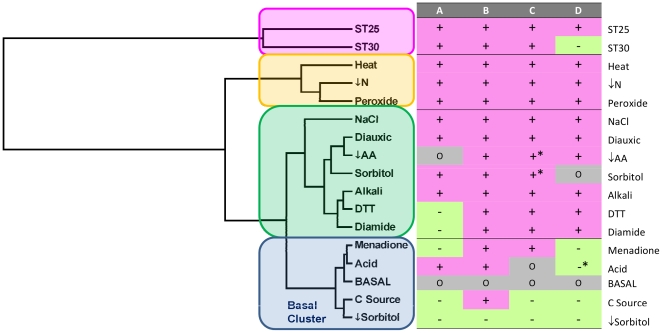
Cluster analysis of the different stress responses. Basal Cluster corresponds to adaptive responses that may occur under energy or resources shortage. Trends in up- and downregulation of genes after stress. (A) Upregulation trend with respect abundance, (B) Downregulation trend with respect abundance, (C) Upregulation trend with respect length, (D) Downregulation trend with respect length. In each case, a (+) result indicates a significant result in the expected direction, (−) means a significant result opposite to the expected one, (o) indicates a non-significant result in the Mann-Whitney analysis. All correlations shown here have p<0.05 and p≤0.06 if *.

A cluster analysis of the twenty five dimensional vectors built for each adaptive response with the index 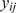 calculated for each GO category is shown in Figure 3. Four clusters can be distinguished from this analysis. Responses to ↓Sorbitol, C source, Menadione and Acid cluster together with basal conditions (*Basal Cluster*) and apart from the other responses. Interestingly, this Basal Cluster includes stress responses in which the previous analytical methods find a low correlation between protein cost and changes in gene expression (Tables 1–2 and Figures 1–2 and S1). Because we could not find an accurate bootstrap statistical test to calculate significance for the clusters in Figure 3 we further tested similitude between the conditions using a discriminant analysis of the data used to build the clusters. Two dimensions explain 99.9% of the variance in the data and separate all four groups found in the cluster analysis (Figure S4).

**Figure 4:**
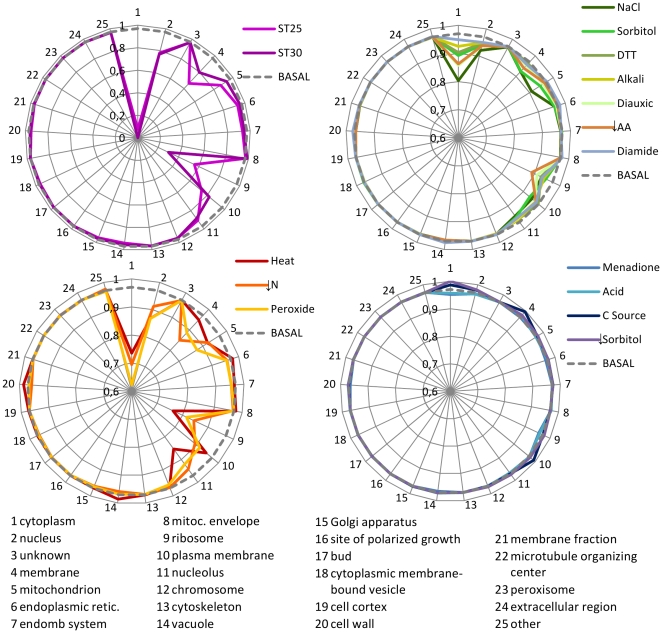
Comparison of the distribution of biosynthetic cost estimates 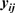 among cellular component GO categories for the various stresses. The values are normalized so that the maximum calculated value of the index in the whole dataset is 1 and the minimum is 0. The basal condition is rescaled to 0.97 and would plot as a circle.

The normalized values of each component of the vector 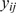 for each type of adaptive response plotted in Figure 4 show the similarity between the different responses. For reference purposes, the basal condition is represented by a dashed circle in each of the panels of that figure. Lines below that circle indicate negative values for 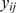 while any lines above the basal condition circle indicate positive values for 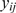. Conditions included in the Basal Cluster show low absolute values for this index in all GO categories. This is different for the other groups that, overall, have larger negative values for 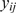 in categories “Cytoplasm”, “Nucleus”, and “Ribosome”. The four clusters are also consistent with the gradation observed in the moving-quantile plots for length and abundance (Figure 2 and S1).

Altogether, the results presented in this section, suggest two broad types of adaptive responses. In one type, corresponding to responses in the Basal Cluster, the changes in gene expression are small. In this group of responses, we find no correlation between protein properties and gene expression. In the other type of stress, responses have evolved in a way that is consistent with a significant pressure to minimize the metabolic cost of the response.

### Adaptive responses that are consistent with metabolic cost minimization

The previous results suggest that the stress conditions considered can be classified in two broad types with respect to metabolic economy. On one hand, we have the Basal Cluster in Figures 3 and 4. This cluster includes the adaptive responses to Menadione, Acid and the two controls, C Source and ↓Sorbitol. The results do not support a significant pressure by metabolic economy in shaping these responses. On the other hand, all other responses can be clustered into three subgroups. Nevertheless, they all appear to be shaped to some degree by metabolic economy. Therefore, we lump together change-folds for gene expression of all these later stress responses. By doing this we create a data set that has a stronger signal than that found in responses to the individual stresses when we relate properties and gene expression changes. The stronger signal in this combined data set also allows us to analyze patterns within each GO category for function, biological processes and cellular location of the proteins.

#### Consistency between the pooled dataset and the individual datasets

As a control for the adequacy of the lumped dataset, we need to make sure that it has the same characteristics as those of its individual constituent datasets. To do this, we compared the gene expression changes between groups of proteins with high and low values for each property in the lumped set of responses (Table 3). This analysis is similar to that described for individual stress responses.

**Table 3:** Comparison between changes in gene expression and different protein properties.

Properties	Up- CF			Down- CF			Thresholds	
	z		p	Z		p	Lower	Upper
Molecular Weight	+	8.68	***	+	5.29	***	46.95	75.23
Length	+	8.47	***	+	5.62	***	414.33	663.67
Pr Abundance	+	6.16	***	+	20.88	***	6.99	13.33
Pr Half-live (T(1/2))	+	0.48	0.32	+	3.42	***	69.67	125.33
Isoelectric Point	−	0.87	0.19	−	0.76	0.22	6.52	8.40
CAI	+	3.41	***	+	21.76	***	0.17	0.24
CodonBias	+	1.22	0.11	+	20.31	***	0.11	0.23
FOP	+	1.49	0.07	+	20.53	***	0.47	0.54
GRAVY	−	1.76	***	+	4.69	***	−0.62	−0.35
Aromaticity	−	4.06	***	−	3.42	***	0.07	0.10
ACPA	−	1.06	0.15	−	3.08	***	22.99	24.02
[mRNA]A	+	4.82	***	+	13.55	***	2.56	4.29
[mRNA]H	+	5.20	***	+	19.80	***	2.07	3.83

Lower and Upper Thresholds indicates the cutoff limits for selecting proteins with low and high values for each of the protein properties. (+) z Indicates that proteins in the Lower group present higher up-expression and lower down-expression than those in the Upper group as compared by the Mann-Whitney analysis. (−) indicates the opposite result.

**Figure 5:**
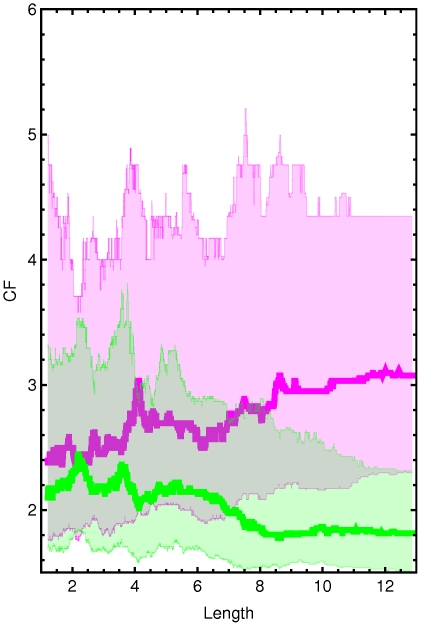
Change-folds of genes in the lumped stress responses with respect to their length and abundance. The plot is the result of moving-quantile 0.75, 0.5 and 0.25 with a window of 300 elements. Green for up-expressed genes and purple for down-expressed. Length is divided by 10^2^ amino acids.

We confirm a strong trend to repress highly abundant proteins and upregulate only proteins that were less abundant under basal conditions. As expected, the other properties follow a coherent pattern that depends on their correlation with abundance (Table S1). For example, highly abundant proteins tend to have high values for mRNA abundance, high codon adaptation indexes, high T_1/2_, low ACPA, and low aromaticity. Consequently, the more repressed proteins also show these traits; the reverse is valid for upregulation. As is the case with the individual datasets, the relationship between changes in gene expression and length is inverse to that one would expect if such a relationship was just a result of the correlation between protein length and abundance. Figure 5 and Table 3 show the tendency to downregulate longer proteins and upregulate shorter proteins during stress response.

An interesting result of this lumped analysis is that both upregulation and downregulation of genes are inversely correlated with CAI. CAI is a proxy for the rate of protein synthesis. This suggests that rate of protein synthesis (affected by CAI) may not be a significant pressure in shaping the responses we are studying. However, it must be stressed that we use CAI (or CB or FOP) estimates for the basal state. These measurements indicate adaptation to the basal tRNA complement of the cell. This complement is likely to change under varying conditions. Therefore, until genome-wide estimates of CAI during adaptive responses are available, rate of protein synthesis cannot be definitively excluded as an important selective pressure in shaping stress responses.

#### Explaining the relationship between length and abundance

As stated earlier, results for length and abundance appears to be counterintuitive if one considers that a) large proteins are not abundant under basal conditions, and yet they tend to be more strongly repressed than short proteins, and b) short proteins are abundant under basal conditions, and yet they tend to be more up-expressed than large proteins.

**Figure 6:**
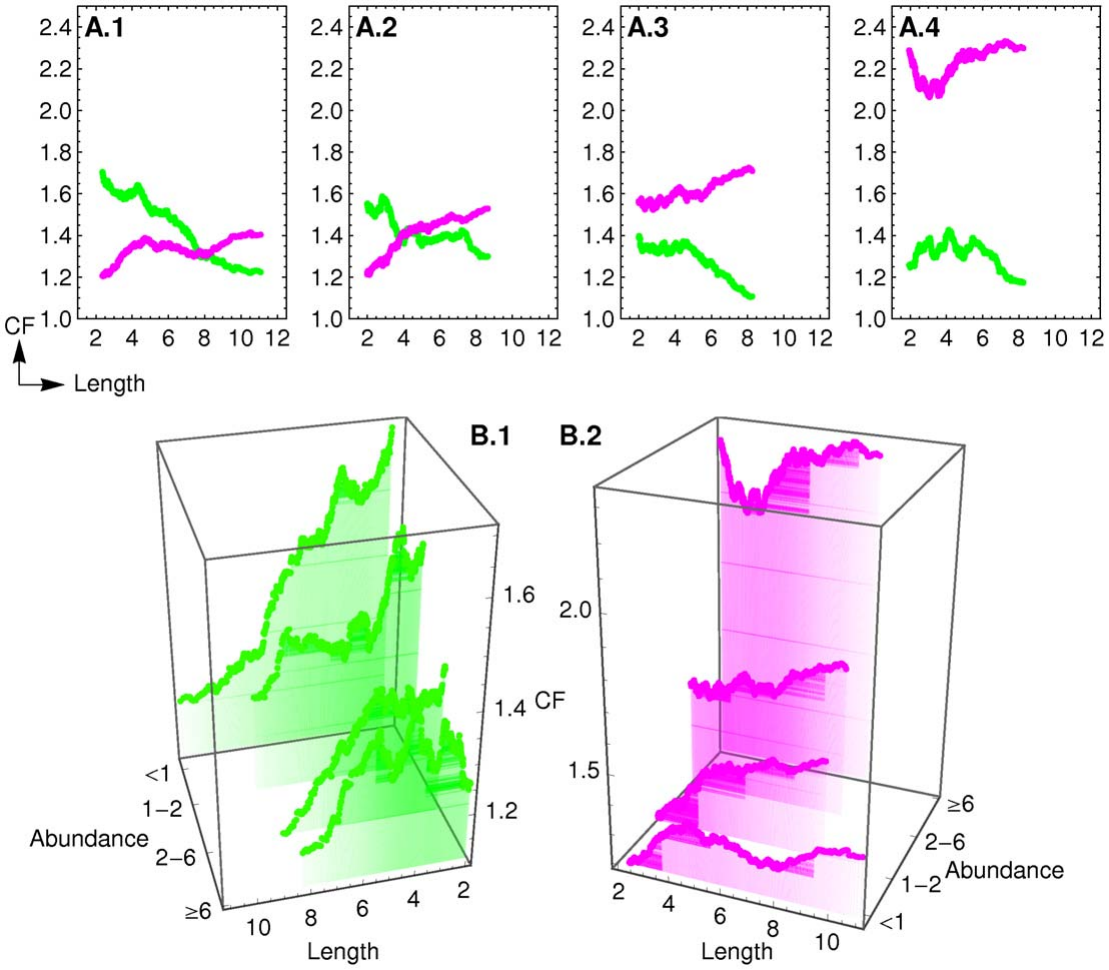
Change-folds of gene expression with respect to their length, binned by their basal abundance. Moving-median plots were calculated using a window of 300 elements. Green - upregulated genes; Purple - downregulated genes. (A) Plot by bins of abundance: (A.1) for proteins with abundance <876 protein per cell, (A.2) abundance between 876 and 2253, (A.3) abundance between 2253 and 6232, and (A.4) if abundance is ≥6232, (B) Shows the results for all bins separated by upregulation (B.1) and downregulation (B.2). Length unit is 10^2^ amino acids and Abundance unit is 10^3^ pr/cell.

Figure 6 provides the clue for understanding the apparent paradox. By plotting gene expression changes with the two properties at the same time one realizes that, in the set of repressed proteins, the longer proteins are more repressed than the shorter ones, while in the set of upregulated proteins, the shorter proteins are more upregulated than the longer ones.

Also, by dividing proteins into four different bins of basal abundance, Figure 6 better illustrates how can protein length be negatively correlated to upregulation of gene expression and positively correlated to downregulation of gene expression during stress response. In summary, within each bin of abundance, short proteins are more upregulated than long proteins and long proteins are more downregulated than short proteins. Moreover, the figure also shows that the maximum size of proteins in a given bin is inversely correlated to the abundance of each bin.

#### Gene Ontology categories

The Mann-Whitney analysis was also performed for the proteins classified in each GO category (for function, process and cellular component). This helps to evaluate if the energetic constraints to gene expression are a general pattern and allows us to control if specific sets of proteins, with a common GO category, contribute very significantly to the observed correlations.

Because each GO category contains a much lower number of proteins than the whole genome, the impact of the noise can be bigger. Even so, Table 4 still shows a statistically significant upregulation of shorter proteins and downregulation of the longer ones in 30 out of 44 cases (68%). In 9 cases no significance was attained and in 5 the results were significantly in the opposite direction.

**Table 4:** Comparison of changes in gene expression between short and large proteins for different functional Super GO-Slim categories.

Category	Up- CF			Down- CF			Thresholds	
	z		p	Z		p	Lower	Upper
**Function**								
Molecular function unknown	+	4.73	***	+	2.98	***	323	548
Catalytic activity	+	5.36	***	+	2.30	***	493	767
Transporter activity	+	1.62	0.05	+	2.69	***	435	710
Structural molecule activity	+	1.47	0.07	−	2.54	***	351	595
Transcription regulator activity	+	1.70	***	+	3.19	***	480	738
Other	+	3.82	***	+	2.27	***	447	730
**Process**								
Cellular physiological process	+	7.18	***	+	3.70	***	443	713
Metabolism	+	5.33	***	+	2.75	***	425	689
Biological process unknown	+	0.03	0.49	+	0.53	0.30	306	505
Transport	+	2.72	***	+	4.18	***	480	773
Transcription	+	1.66	***	+	2.61	***	499	792
Cell cycle	+	3.10	***	+	2.37	***	543	843
Amino acid metabolism	+	1.23	0.11	+	1.10	0.14	467	674
Signal transduction	+	2.40	***	+	1.33	0.09	548	885
Other	+	2.16	***	−	0.97	0.17	385	642
**Component**								
Cytoplasm	+	5.67	***	+	4.08	***	416	672
Nucleus	+	6.10	***	+	3.66	***	462	743
Cellular component unknown	−	4.07	***	−	1.86	***	275	446
Mitochondrion	+	5.77	***	+	7.59	***	436	719
Endoplasmic reticulum	+	2.29	***	+	2.32	***	384	597
Cytosol	−	2.83	***	−	6.73	***	306	505
Other	+	3.85	***	+	0.24	0.40	473	741

To further investigate the negative results, we analyzed both the basal abundance and the frequency of proteins involved in molecular complexes for each category (Table 5). Categories “Structural molecule activity” and “Cytosol” have the highest percentage of proteins that are involved in molecular complexes, 0.83 and 0.53 respectively. The GO category “Cellular component unknown” is the group with lowest abundance and also with the lowest thresholds for the Mann-Whitney analysis which means that this group is composed of short proteins.

**Table 5:** Categorization by Super Go-Slim: Molecular complexes and protein concentrations.

Category	Complexes		Protein Abundance			
	N	Freq	Mean	0.25	0.5	0.75
**Function**						
Molecular function unknown	137	0.05	4.31	0.72	1.71	3.42
Catalytic activity	392	0.20	15.90	1.18	3.04	8.36
Transporter activity	86	0.21	18.71	0.91	2.97	8.50
Structural molecule activity	280	***0.83***	30.17	1.82	6.22	31.59
Transcription regulator activity	134	0.41	3.05	0.54	1.36	3.51
Other	307	0.34	11.98	0.86	2.18	6.14
**Process**						
Cellular physiological process	1239	0.28	13.89	1.04	2.58	7.08
Metabolism	1059	0.35	16.01	1.17	2.87	7.82
Biological process unknown	14	0.01	3.11	0.59	1.44	3.26
Transport	201	0.21	12.69	1.11	2.75	6.92
Transcription	246	0.49	4.28	0.77	1.73	4.49
Cell cycle	140	0.34	4.00	0.53	1.38	3.69
Amino acid metabolism	19	0.10	30.86	2.02	6.90	26.52
Signal transduction	13	0.07	5.68	0.72	1.52	3.95
Other	1	0.01	3.43	0.52	1.44	5.54
**Component**						
Cytoplasm	670	0.20	14.04	1.08	2.73	7.39
Nucleus	664	0.35	7.94	0.91	2.25	5.41
Cellular component unknown	2	0.00	***1.94***	***0.34***	***0.81***	***1.80***
Mitochondrion	242	0.24	10.31	1.08	2.54	6.86
Endoplasmic reticulum	30	0.09	10.54	1.21	2.84	6.76
Cytosol	188	***0.58***	45.80	3.46	13.67	52.33
Other	86	0.15	12.05	0.63	1.73	6.07

For each group we computed the number (N) and frequency of genes in molecular complexes, and the mean and quartiles of protein concentrations.

We also made the analysis using more detailed GO terms. As shown in Supplementary Tables S2, S3, S4, 87 out of 164 cases are in concordance with our previous results in a statistically significant way, while only 7 cases (in six GO categories) go against the hypotheses. No statistical significance could be obtained for the remaining GO categories. Trends between changes in gene expression and protein size are general and not specific to a few categories. Categories that we would like to remark and in which the relationship between changes in gene regulation and protein properties is consistent with our hypotheses are: “Molecular function unknown”, “Hydrolase activity”, “Transport”, “Protein modification”, “Protein catabolism”, “DNA metabolism”, “RNA metabolism”, and “Response to stress”.

One of the six categories in which that relationship is inconsistent with the hypotheses is “Ribosome”. The other five categories are “Structural molecule activity”, “Helicase activity”, “Sporulation”, “Molecular function unknown”, and “Cellular component unknown”. We could expect that ribosomal proteins would contribute strongly to the hypothesized trends because they are highly abundant under basal conditions and highly repressed during stress. However, the results discard that those proteins are a major contributor for the general trends observed for the whole genome.

Several factors may explain the exceptions for some GO categories. First, the category may include mostly proteins whose specific function is required for the response. Such a situation could overcome a pressure for economy in protein synthesis. Interestingly, the consistency of the “Response to stress” category with our hypothesis suggests that such cases may be rare. Second, the relevant category may contain a high proportion of genes that code for proteins of very low basal abundance. Because the proteins in these groups contribute poorly, if at all, to the total cell mass, one could expect that the selective pressure for economy in protein synthesis is weak. Third, a high proportion of genes in a functional group may be involved in complexes. Whenever a GO category contains more than 50% of genes that are involved in molecular complexes, no correlation is found between protein length and gene expression changes (Tables 5, S5, S6, S7). GO categories that fit this description are: “Structural molecule activity” (83%), “Cytosol” (58%), “Translator regulator activity” (71%), “Motor activity” (56%), “Protein biosynthesis” (64%), “Electron transport” (70%), and “Ribosome” (99%). The selective pressure for economy in protein synthesis should impinge more strongly on the complexes themselves than on the individual proteins.

#### Proteins involved in complexes

Understanding if and how the size and abundance of protein in complexes is affected by a pressure to save metabolic costs in protein synthesis would require taking into account the size of the individual complexes. This, in turn, requires that the stoichiometry of those complexes is known with confidence. Because this information is not available for most protein complexes of yeast, a detailed analysis must await accurate data regarding such stoichiometry.

In *S. cerevisiae*, about 1500 proteins participate in molecular complexes [30],[31]. This is about one third of all proteins coded by the yeast genome. It is possible (even likely) that proteins involved in the formation of a given complex have coordinated regulation of gene expression [32]. If this is so, the cost of changing the expression of the complex should take into account the size of each of the subunits and its corresponding stoichiometry [33]. Thus, one might expect that selective pressures that regulate the evolution of gene expression act similarly in the groups of genes coding for proteins that form a complex and in genes coding for large proteins. Consequently, if a transcriptional profile has evolved under conditions of resource and energy scarcity, the genes coding for complexes are expected to be more strongly repressed during stress than genes coding for proteins that are not involved in complexes.

To test this hypothesis in the absence of data about stoichiometry of each complex, we selected genes coding for proteins that are flagged in SGD as being part of a protein complex. The analysis confirms that genes coding for proteins involved in complexes are more strongly repressed than other genes (z = 9.46, p<0.05).

**Figure 7:**
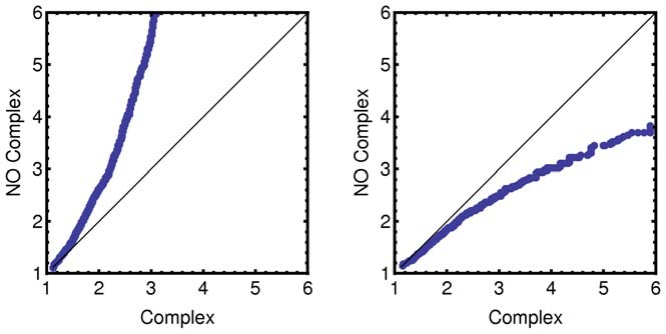
Comparison of the change-fold between proteins that are part of a complex and those that are not. Quantile-quantile plots show the divergence between the two lists by the deviation of the points from the line with a slope of 1. (A) Tendencies of the up-expression change-folds; (B) Tendencies of the down-expression change-folds.

Similarly, genes coding for proteins involved in complex formation are less upregulated than those coding for proteins not involved in complex formation (z = 16.22, p<0.05). This can be seen in the quantile-quantile plots of the change-fold shown in Figure 7.

## Discussion

What type of general selective pressure might explain the correlations we find between changes in gene expression and protein abundance or length during stress response? One answer to this question is that minimizing the cost of protein synthesis is a significant pressure that shapes changes in gene expression during adaptive responses. Why would minimizing metabolic costs improve fitness of *S. cerevisiae*? As the cell optimizes the expenditure of resources for metabolic maintenance, it will have more resources available for survival and reproduction, thus out competing organisms. This seem logical, but it also raises another question, which is why would one expect this pressure to be felt at the level of proteins?

Calculations based on the typical cellular composition of yeast and bacteria predict that protein synthesis uses more metabolic resources and ATP molecules than the formation of other macromolecules and it is a limiting step for yield [12],[34],[35],[36],[37]. As proteins of a shorter size use less amino acids, evolving fully functional short proteins leads to faster protein synthesis with less usage of cellular resources. It must be stressed that this argument cannot be seen as defending that cell will, over time, simply loose all large proteins and use smaller proteins to perform all necessary molecular functions. Evolution is constrained by life history. Specifically, the evolutionary unit of proteins is the functional domain [38]. Such functional domains have on average appeared only once in evolution and examples of domain convergent evolution are rare. Protein function often depends on how a small number of amino acids are located within the 3D structure of these domains. Therefore, a shorter protein may not have a 3D structure that will allow for the maintenance of an appropriate biological activity. This will constrain the amount of resources that can be saved by evolving shorter proteins.

Under stress, availability of resources may be significantly limited, and the cell must adapt quickly in order to survive. For challenging stress conditions, resource limitation may impose severe limitations to the adaptive response. Exposure to these kinds of stresses causes the cell to deviate considerable resources from its steady state metabolism towards the adaptive response and imposes important constraints to cell economy [11],[39]. For example during exposure to high NaCl concentrations, additional energy expenditure for growth increased between 14% and 31% [40], and the activity of the plasma membrane H^+^-ATPase (highest consumer of ATP) is repressed during heat shock or in the presence of a weak acid [11],[41]. Another situation that has been put forward as supporting a cellular energetic shortage during stress response is the hypersensitivity to oxidative stress of mutants that lack mitochondrial function and of yeast treated with mitochondrial inhibitors [42]. These authors suggest that the oxidative sensitivity is due to a defect in an energy-requiring process that is needed for detoxification of ROS or for the repair of oxidative molecular damage.

Further support for the importance of protein cost as a selective pressure in the evolution of adaptive changes in gene expression is found in different studies. For example, pathways appear to have evolved to maximize flux for a minimum amount of protein, because the enzyme concentration may be limited by both the protein synthesizing capacity and the solvent capacity of a cell [43]. In fact, theoretical studies suggest that adaptive responses of yeast to environmental changes trigger a gene expression profile that is optimal under the constraint of minimal total enzyme production [2],[44],[45].

There are three aspects that the cell can tune to decrease cost of protein synthesis. First, it can decrease the amount of protein that it synthesizes per time units. If we take changes in gene expression as a proxy of changes in protein synthesis, we find that, in many cases the overall protein synthesis during stress response is decreased (the y_ij_ index defined above is negative). Second, the cell may decrease cost of protein synthesis by expressing at higher levels proteins that are small. This would decrease the biosynthetic cost per protein chain and is consistent with our results. Finally, the cell may decrease the cost of protein synthesis by increasing the half life of proteins. We find no evidence for this strategy.

In summary, if decreasing the cost of protein synthesis significantly contributes to shaping the gene expression profile of an adaptive response, we should find trends in the composition of the changing protein complement that are consistent with the following predictions:

- **Downregulation of genes that are highly expressed under normal conditions and thus code for highly abundant proteins**. By repressing these genes, the cell can significantly save resources that can then be used in the stress response [46]. For example, ribosomal proteins make for a large fraction of a cell's protein complement, and the resources invested in keeping pools of ribosomal proteins are high [47]. It is well known that the expression of ribosomal genes is significantly repressed under many different stress conditions.
- **Upregulation will preferably occur in genes that have low expression levels under normal conditions**. Our results support this prediction.
  Because long proteins are more expensive to make than small proteins, protein length is an important component of the cost of protein synthesis. If cost of protein synthesis is minimized during the response we would expect that:
- **Downregulation of genes that code for large proteins**. This is so because such a pattern would save resources to the cell.
- **Upregulation will be found preferably in the expression of genes that code for small proteins**. This would save resources and allow for a faster protein synthesis.

The results of our analysis are broadly consistent with these predictions (see Figure 3 for a summary) and support the hypothesis that response to the various stresses has evolved under a selective pressure for minimizing the cost of protein synthesis. GO analysis show that the results are not biased by a specific type of proteins and that the hypotheses are consistent with the results over a wide variety of GO categories. We also see that proteins involved in molecular complexes have changes in gene expression that are similar to proteins that are very large. A more detailed analysis of this later result would require an accurate knowledge about the stoichiometry of the complexes.

Further analysis that would directly establish whether there are limitations on resources and energy usage during a given adaptive response would require data about ATP usage and production under each relevant condition. Such data would allow us to better understand which constraints are important in shaping the evolution of those responses.

## Supporting Information

Figure S1 Change-folds of genes with respect to basal abundance. Plots show the moving-quantiles using a window of 300 elements. Colors: Green for upregulation and purple for downregulation. Abundance unit is 10^4^ pr/cell.
(0.35 MB TIF)

Figure S2 Change-folds of genes with respect to protein half-live. Plots show the moving-quantiles using a window of 300 elements. Colors: Green for upregulation and purple for downregulation.
(0.40 MB TIF)

Figure S3 Change-folds of genes with respect to CAI. Plots show the moving-quantiles using a window of 300 elements. Colors: Green for upregulation and purple for downregulation.
(0.45 MB TIF)

Figure S4 Discriminant analysis. Environmental conditions were classified in four groups: 1) Basal Cluster- Basal vector, menadione, acid, change in carbon source, and sorbitol depletion; 2) NaCl, diauxic, aminoacid depletion, presence of sorbitol, akali, DTT, diamide; 3) heat shock, peroxide, nitrogen depletion; 4) stationary phase at 25°C and 30°C.
(0.07 MB TIF)

Table S1 Spearman Rank Correlation Matrix between different physical properties of genes and proteins. ^0^ Not statistically significant.
(0.08 MB DOC)

Table S2 Comparison of changes in gene expression between short and large proteins for different functional Yeast GO Slim categories.
(0.05 MB DOC)

Table S3 Comparison of changes in gene expression between short and large proteins for different process Yeast GO Slim categories.
(0.07 MB DOC)

Table S4 Comparison of changes in gene expression between short and large proteins for different cell component Yeast GO Slim categories.
(0.06 MB DOC)

Table S5 Categorization by Function (Yeast Go-Slim): Molecular complexes and protein concentrations. For each group we computed the number and frequency of genes related to any molecular complex, and the mean and quartiles of protein concentrations.
(0.06 MB DOC)

Table S6 Categorization by Process (Yeast Go-Slim Molecular complexes and protein concentrations. For each group we computed the number and frequency of genes related to any molecular complex, and the mean and quartiles of protein concentrations.
(0.07 MB DOC)

Table S7 Categorization by Molecular Component(Yeast Go-Slim): Molecular complexes and protein concentrations. For each group we computed the number and frequency of genes related to any molecular complex, and the mean and quartiles of protein concentrations.
(0.06 MB DOC)
