# Supplementary figures and images for: Minimization of Biosynthetic Costs in Adaptive Gene Expression Responses of Yeast to Environmental Changes
Source: PLoS Comput Biol. 2010 Feb 12;6(2):e1000674. doi: 10.1371/journal.pcbi.1000674 (PMC2820516; doi:10.1371/journal.pcbi.1000674)

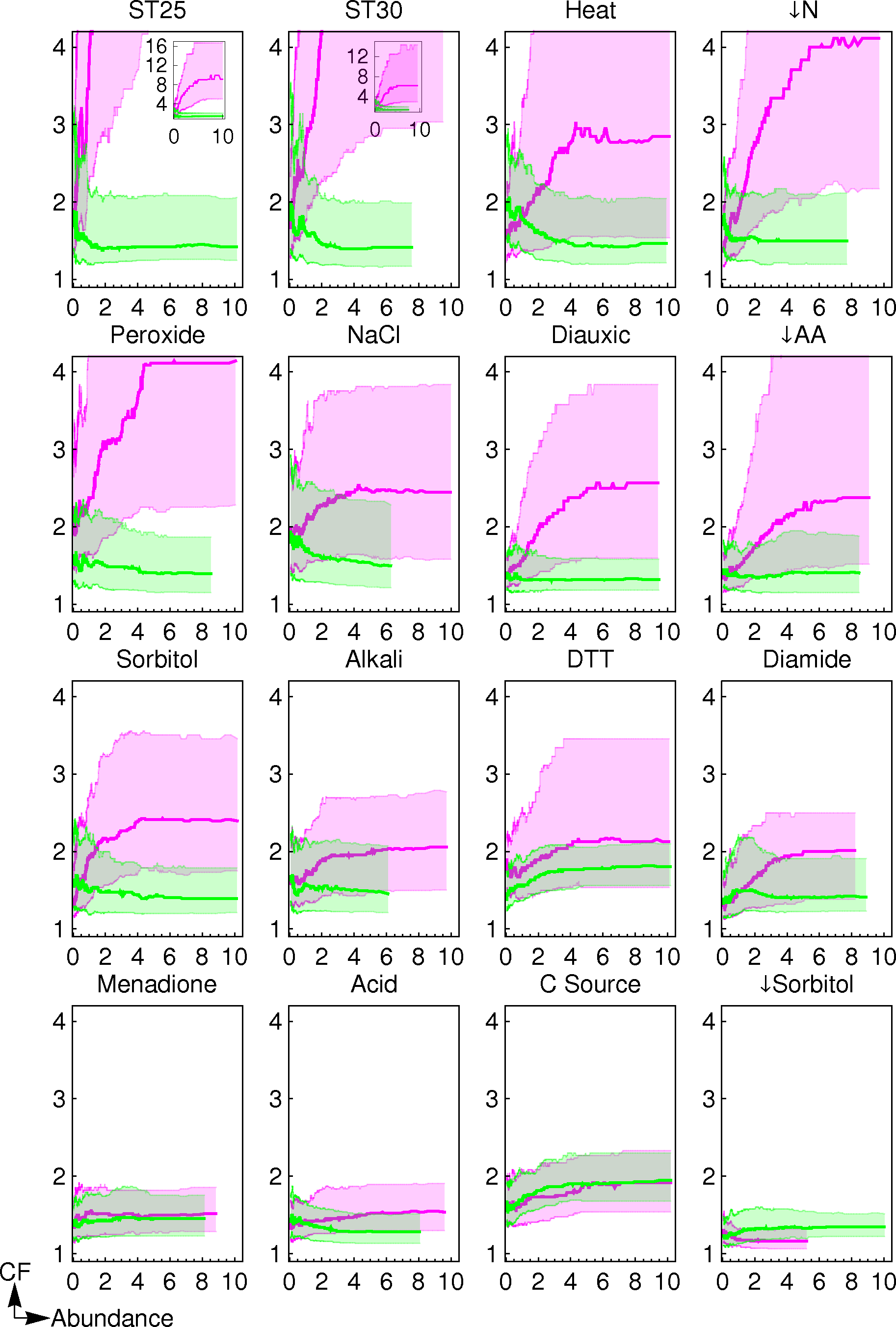

Supplement: Figure S1 — Change-folds of genes with respect to basal abundance. Plots show the moving-quantiles using a window of 300 elements. Colors: Green for upregulation and purple for downregulation. Abundance unit is 104 pr/cell. (0.35 MB TIF) [file pcbi.1000674.s001.tif]

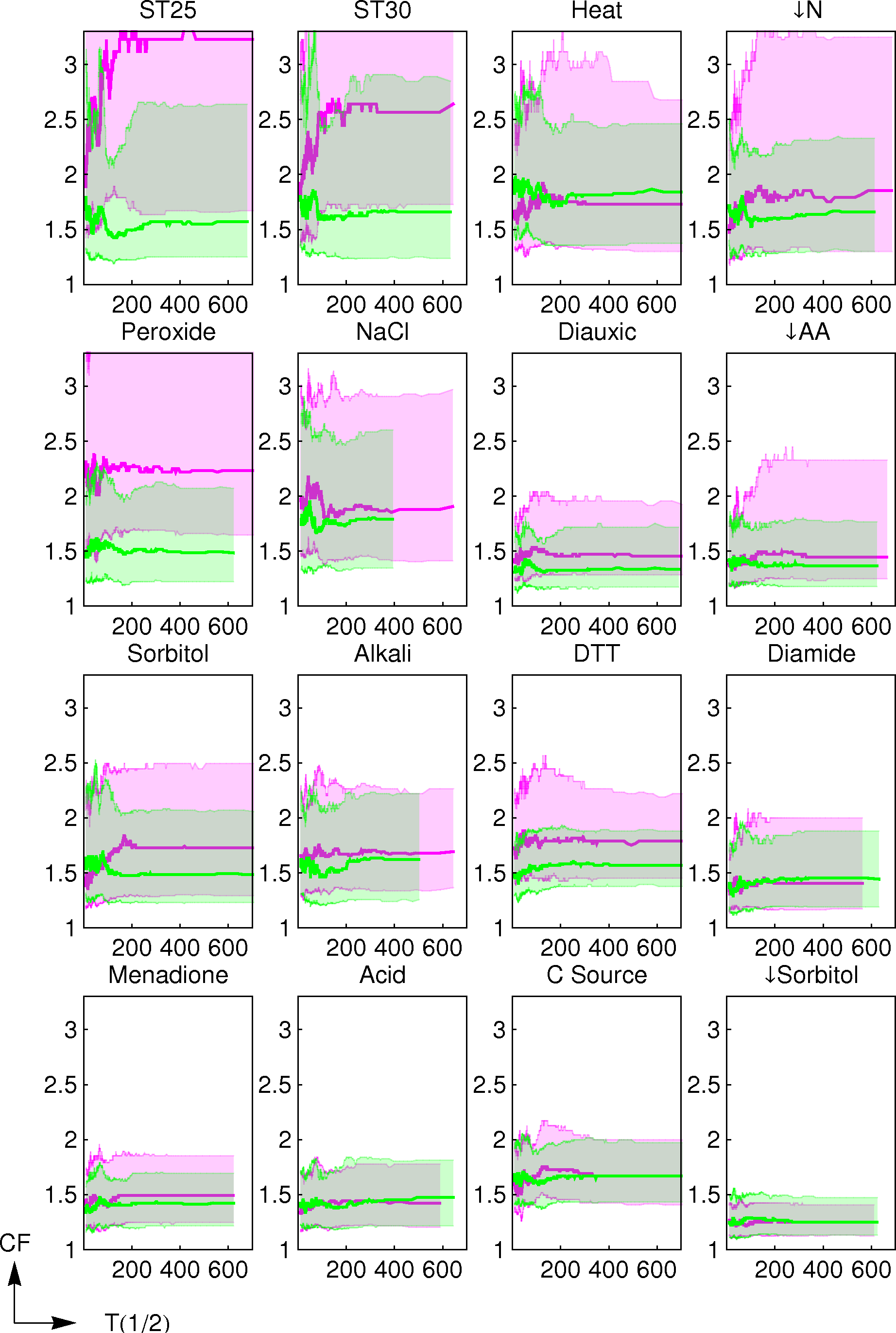

Supplement: Figure S2 — Change-folds of genes with respect to protein half-live. Plots show the moving-quantiles using a window of 300 elements. Colors: Green for upregulation and purple for downregulation. (0.40 MB TIF) [file pcbi.1000674.s002.tif]

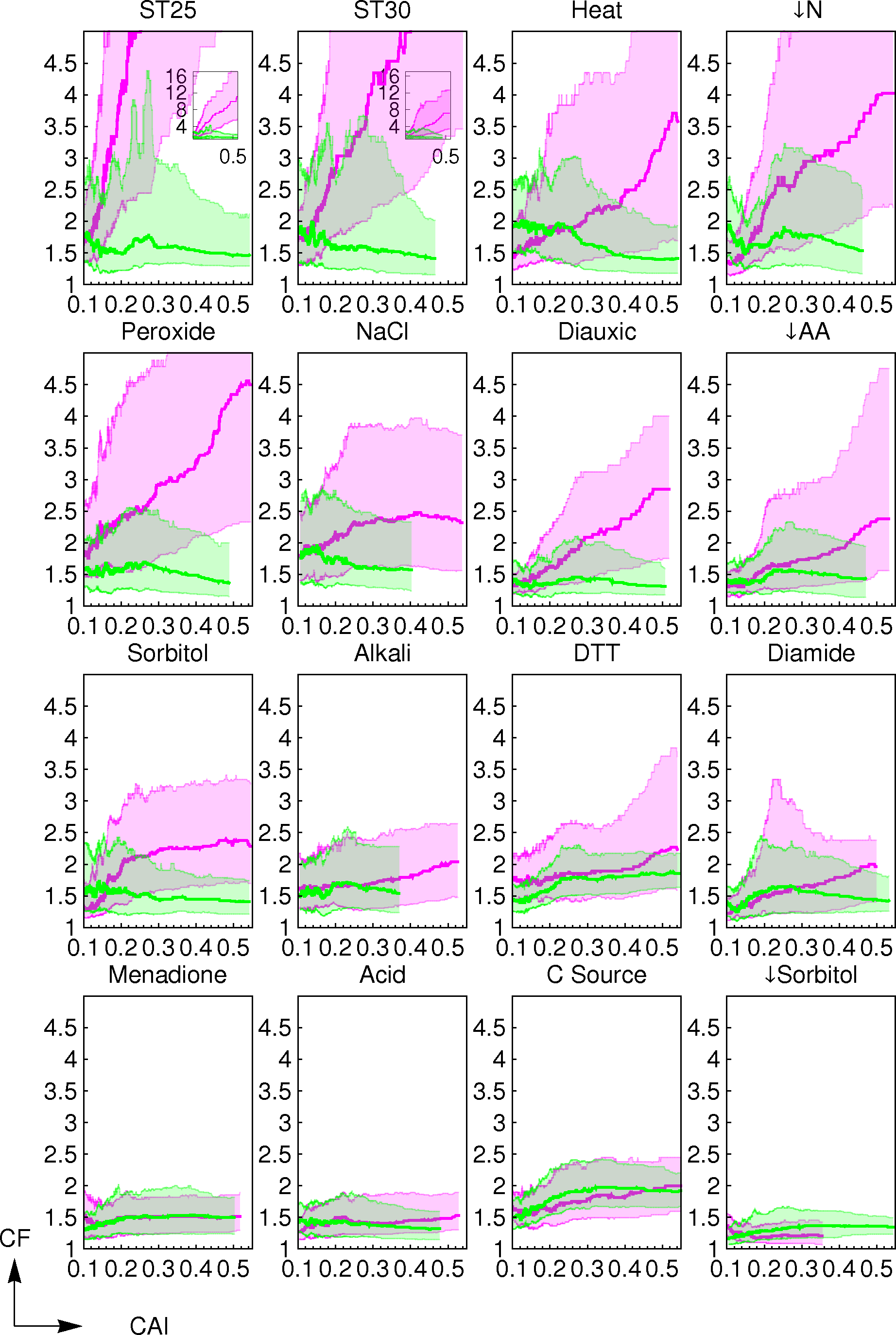

Supplement: Figure S3 — Change-folds of genes with respect to CAI. Plots show the moving-quantiles using a window of 300 elements. Colors: Green for upregulation and purple for downregulation. (0.45 MB TIF) [file pcbi.1000674.s003.tif]

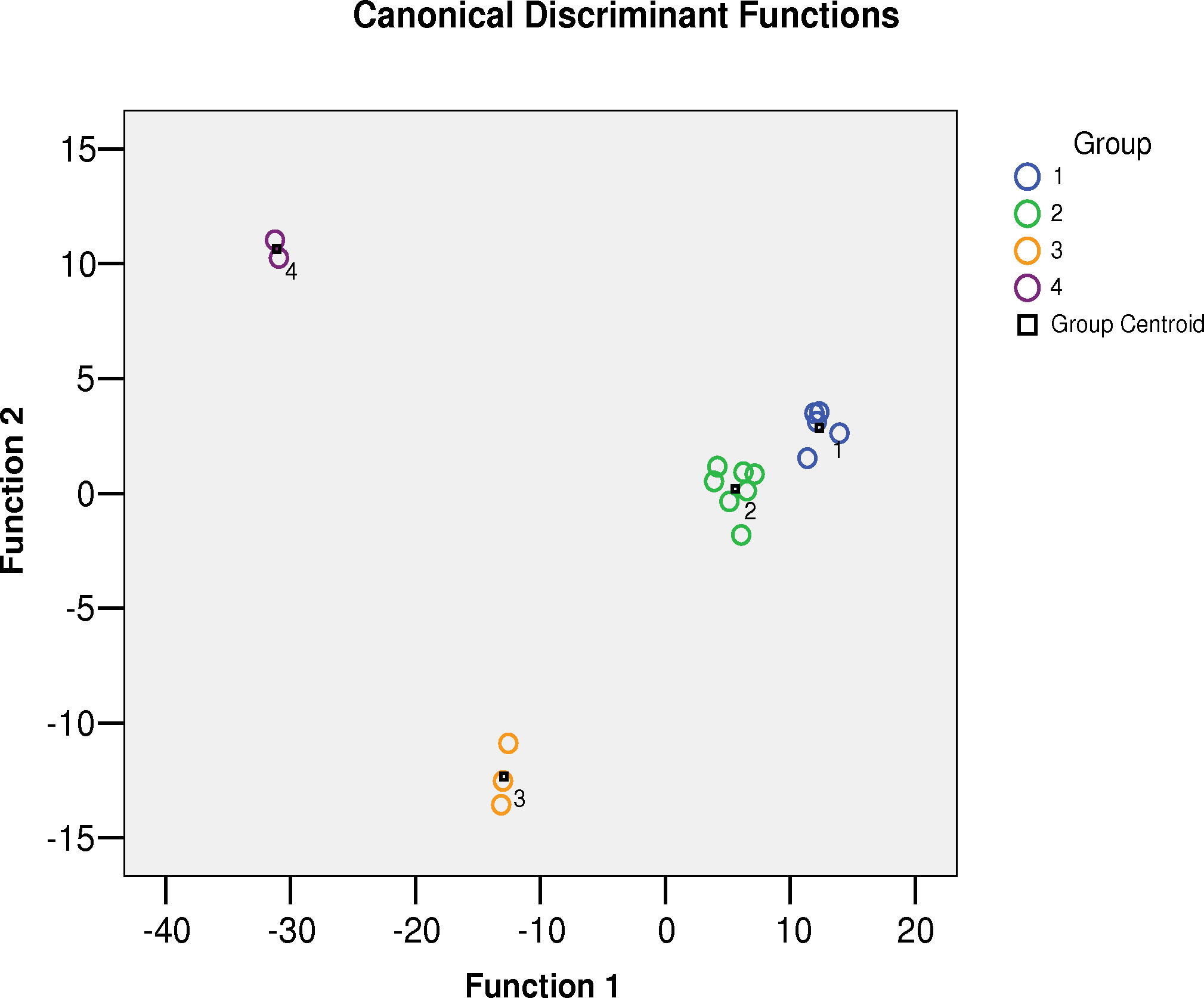

Supplement: Figure S4 — Discriminant analysis. Environmental conditions were classified in four groups: 1) Basal Cluster- Basal vector, menadione, acid, change in carbon source, and sorbitol depletion; 2) NaCl, diauxic, aminoacid depletion, presence of sorbitol, akali, DTT, diamide; 3) heat shock, peroxide, nitrogen depletion; 4) stationary phase at 25°C and 30°C. (0.07 MB TIF) [file pcbi.1000674.s004.tif]
