# Supplementary material for: Minimization of Biosynthetic Costs in Adaptive Gene Expression Responses of Yeast to Environmental Changes
Source: PLoS Comput Biol. 2010 Feb 12;6(2):e1000674. doi: 10.1371/journal.pcbi.1000674 (PMC2820516; doi:10.1371/journal.pcbi.1000674)
Supplement: Table S1 — Spearman Rank Correlation Matrix between different physical properties of genes and proteins. 0 Not statistically significant. (0.08 MB DOC) [file pcbi.1000674.s005.doc]

|  |  |  |  |  |  |  |  |  |  |  |  |  |
| --- | --- | --- | --- | --- | --- | --- | --- | --- | --- | --- | --- | --- |

|  | **MW** | **Length** | **Pr Ab.** | **T_1/2_** | **IP** | **CAI** | **CB** | **FOP** | **GRAVY** | **ARO** | **ACPA** | **[mRNA]A** | **[mRNA]H** |
| --- | --- | --- | --- | --- | --- | --- | --- | --- | --- | --- | --- | --- | --- |
| **MW** | 1 | 1.00 | -0.16 | -0.23 | -0.26 | 0.21 | 0.08 | 0.12 | -0.24 | -0.12 | -0.22 | -0.20 | -0.18 |
| **Length** | 1.00 | 1 | -0.16 | -0.23 | -0.27 | 0.21 | 0.09 | 0.13 | -0.23 | -0.13 | -0.24 | -0.20 | -0.17 |
| **P.Abundance** | -0.16 | -0.16 | 1 | 0.32 | -0.13 | 0.54 | 0.53 | 0.54 | 0.13 | -0.06 | -0.10 | 0.56 | 0.57 |
| **T_1/2_** | -0.23 | -0.23 | 0.32 | 1 | -0.02^0^ | 0.18 | 0.18 | 0.19 | 0.09 | -0.03^0^ | 0.03 | 0.19 | 0.22 |
| **IP** | -0.26 | -0.27 | -0.13 | -0.02^0^ | 1 | -0.25 | -0.14 | -0.19 | 0.05 | 0.11 | 0.33 | 0.05 | -0.06 |
| **CAI** | 0.21 | 0.21 | 0.54 | 0.18 | -0.25 | 1 | 0.83 | 0.89 | -0.14 | -0.14 | -0.23 | 0.64 | 0.53 |
| **CodonBias** | 0.08 | 0.09 | 0.53 | 0.18 | -0.14 | 0.83 | 1 | 0.97 | -0.00^0^ | -0.17 | -0.26 | 0.60 | 0.55 |
| **FOP** | 0.12 | 0.13 | 0.54 | 0.19 | -0.19 | 0.89 | 0.97 | 1 | -0.04 | -0.17 | -0.24 | 0.61 | 0.55 |
| **GRAVY** | -0.24 | -0.23 | 0.13 | 0.09 | 0.05 | -0.14 | -0.00^0^ | -0.04 | 1 | 0.48 | 0.49 | -0.01^0^ | 0.06 |
| **ARO** | -0.12 | -0.13 | -0.06 | -0.03 | 0.11 | -0.14 | -0.17 | -0.17 | 0.48 | 1 | 0.84 | -0.17 | -0.12 |
| **ACPA** | -0.22 | -0.24 | -0.10 | 0.03^0^ | 0.33 | -0.23 | -0.26 | -0.24 | 0.49 | 0.84 | 1 | -0.15 | -0.15 |
| **[mRNA]A** | -0.20 | -0.20 | 0.56 | 0.19 | 0.05 | 0.64 | 0.60 | 0.61 | -0.01 | -0.17 | -0.15 | 1 | 0.70 |
| **[mRNA]H** | -0.18 | -0.17 | 0.57 | 0.22 | -0.06 | 0.53 | 0.55 | 0.55 | 0.06^0^ | -0.12 | -0.15 | 0.70 | 1 |
