# Supplementary material for: Minimization of Biosynthetic Costs in Adaptive Gene Expression Responses of Yeast to Environmental Changes
Source: PLoS Comput Biol. 2010 Feb 12;6(2):e1000674. doi: 10.1371/journal.pcbi.1000674 (PMC2820516; doi:10.1371/journal.pcbi.1000674)
Supplement: Table S2 — Comparison of changes in gene expression between short and large proteins for different functional Yeast GO Slim categories. (0.05 MB DOC) [file pcbi.1000674.s006.doc]

|  |  |  |  |  |  |  |  |  |
| --- | --- | --- | --- | --- | --- | --- | --- | --- |
| **Function** | **Up- CF** | | | **Down- CF** | | | **Thresholds** | |
|  | **z** | | **p** | **z** | | **p** | **Lower** | **Upper** |
| Molecular function unknown | + | 4.73 | *** | + | 2.98 | *** | 330 | 548 |
| Hydrolase activity | + | 2.83 | *** | + | 2.45 | *** | 524 | 817 |
| Transferase activity | + | 1.11 | 0.13 | - | 0.24 | 0.40 | 494 | 756 |
| Protein binding | + | 3.35 | *** | + | 2.73 | *** | 465 | 755 |
| Transporter activity | + | 1.62 | 0.05 | + | 2.69 | *** | 435 | 710 |
| Structural molecule activity | + | 1.47 | 0.07 | - | 2.54 | *** | 351 | 595 |
| Transcription regulator activity | + | 1.70 | *** | + | 3.19 | *** | 480 | 738 |
| RNA binding | + | 1.85 | *** | + | 1.00 | 0.16 | 406 | 665 |
| Oxidoreductase activity | + | 1.67 | *** | + | 4.40 | *** | 331 | 513 |
| DNA binding | + | 2.22 | *** | + | 0.12 | 0.45 | 471 | 724 |
| Enzyme regulator activity | + | 1.56 | 0.06 | + | 0.56 | 0.29 | 501 | 828 |
| Peptidase activity | + | 1.50 | 0.07 | + | 0.71 | 0.24 | 488 | 741 |
| Protein kinase activity | + | 1.04 | 0.15 | + | 2.03 | *** | 655 | 937 |
| Nucleotidyltransferase activity | + | 1.62 | 0.05 | - | 0.06 | 0.48 | 513 | 883 |
| Ligase activity | + | 0.46 | 0.32 | + | 0.27 | 0.39 | 611 | 895 |
| Helicase activity | - | 2.08 | *** | - | 4.45 | *** | 841 | 1182 |
| Lyase activity | - | 0.52 | 0.30 | + | 0.35 | 0.36 | 439 | 608 |
| Signal transducer activity | - | 0.41 | 0.34 | - | 1.16 | 0.12 | 561 | 890 |
| Translation regulator activity | + | 0.82 | 0.21 | - | 0.42 | 0.34 | 429 | 697 |
| Isomerase activity | + | 0.88 | 0.19 | + | 0.18 | 0.43 | 355 | 528 |
| Phosphoprotein phosphatase activity | + | 0.12 | 0.45 | + | 0.93 | 0.17 | 412 | 615 |
| Motor activity | + | 0.77 | 0.22 | + | 2.09 | *** | 742 | 1335 |
| Other | + | 1.73 | *** | + | 1.56 | 0.06 | 492 | 830 |
