# Supplementary material for: Minimization of Biosynthetic Costs in Adaptive Gene Expression Responses of Yeast to Environmental Changes
Source: PLoS Comput Biol. 2010 Feb 12;6(2):e1000674. doi: 10.1371/journal.pcbi.1000674 (PMC2820516; doi:10.1371/journal.pcbi.1000674)
Supplement: Table S3 — Comparison of changes in gene expression between short and large proteins for different process Yeast GO Slim categories. (0.07 MB DOC) [file pcbi.1000674.s007.doc]

| **Process** | **Up- CF** | | | **Down- CF** | | | **Thresholds** | |
| --- | --- | --- | --- | --- | --- | --- | --- | --- |
|  | **z** | | **P** | **z** | | **p** | **Lower** | **Upper** |
| Unknown | + | 0.03 | 0.49 | + | 0.53 | 0.30 | 306 | 505 |
| Organelle organization and biogenesis | + | 2.32 | *** | - | 0.32 | 0.37 | 474 | 781 |
| Transport | + | 2.72 | *** | + | 4.18 | *** | 480 | 773 |
| Protein modification | + | 2.58 | *** | + | 2.24 | *** | 444 | 685 |
| Transcription | + | 1.66 | *** | + | 2.61 | *** | 499 | 792 |
| Protein biosynthesis | + | 2.21 | *** | - | 1.39 | 0.08 | 333 | 546 |
| DNA metabolism | + | 2.34 | *** | + | 2.55 | *** | 519 | 831 |
| RNA metabolism | + | 4.76 | *** | + | 3.47 | *** | 446 | 708 |
| Response to stress | + | 4.15 | *** | + | 1.87 | *** | 489 | 811 |
| Cell cycle | + | 3.10 | *** | + | 2.37 | *** | 543 | 843 |
| Ribosome biogenesis and assembly | + | 4.29 | *** | + | 0.90 | 0.18 | 430 | 685 |
| Vesicle-mediated transport | + | 2.09 | *** | + | 2.09 | *** | 536 | 877 |
| Morphogenesis | + | 1.68 | *** | - | 0.16 | 0.44 | 599 | 983 |
| Generation of precursor metabolites and energy | - | 0.51 | 0.30 | + | 2.08 | *** | 360 | 594 |
| Lipid metabolism | + | 1.84 | *** | - | 0.67 | 0.25 | 541 | 803 |
| Cytoskeleton organization and biogenesis | + | 0.73 | 0.23 | + | 0.38 | 0.35 | 561 | 931 |
| Carbohydrate metabolism | + | 0.49 | 0.31 | + | 0.13 | 0.45 | 508 | 761 |
| Amino acid and derivative metabolism | + | 1.07 | 0.14 | + | 0.77 | 0.22 | 481 | 680 |
| Signal transduction | + | 2.40 | *** | + | 1.33 | 0.09 | 548 | 885 |
| Protein catabolism | + | 2.00 | *** | + | 2.07 | *** | 418 | 631 |
| Cell wall organization and biogenesis | - | 0.78 | 0.22 | - | 1.40 | 0.08 | 569 | 909 |
| Meiosis | + | 2.05 | *** | + | 0.31 | 0.38 | 539 | 820 |
| Cell homeostasis | + | 0.55 | 0.29 | - | 0.69 | 0.24 | 403 | 642 |
| Sporulation | + | 0.86 | 0.20 | - | 2.22 | *** | 429 | 649 |
| Conjugation | + | 1.77 | *** | - | 0.85 | 0.20 | 465 | 756 |
| Cytokinesis | + | 0.43 | 0.33 | - | 1.38 | 0.08 | 556 | 946 |
| Membrane organization and biogenesis | + | 0.93 | 0.18 | + | 2.61 | *** | 428 | 738 |
| Cellular respiration | + | 0.72 | 0.24 | + | 2.43 | *** | 289 | 461 |
| Cell budding | - | 0.20 | 0.42 | - | 0.12 | 0.45 | 592 | 1020 |
| Vitamin metabolism | + | 0.38 | 0.35 | - | 0.50 | 0.31 | 378 | 533 |
| Pseudohyphal growth | + | 2.29 | *** | - | 0.40 | 0.34 | 514 | 760 |
| Nuclear organization + biogenesis | + | 1.24 | 0.11 | + | 2.10 | *** | 633 | 1047 |
| Electron transport | + | 0.07 | 0.47 | + | 1.33 | 0.09 | 241 | 416 |
| Other | - | 0.35 | 0.36 | + | 0.72 | 0.24 | 365 | 573 |
