# Supplementary material for: Minimization of Biosynthetic Costs in Adaptive Gene Expression Responses of Yeast to Environmental Changes
Source: PLoS Comput Biol. 2010 Feb 12;6(2):e1000674. doi: 10.1371/journal.pcbi.1000674 (PMC2820516; doi:10.1371/journal.pcbi.1000674)
Supplement: Table S4 — Comparison of changes in gene expression between short and large proteins for different cell component Yeast GO Slim categories. (0.06 MB DOC) [file pcbi.1000674.s008.doc]

|  |  |  |  |  |  |  |  |  |
| --- | --- | --- | --- | --- | --- | --- | --- | --- |
| **Component** | **Up- CF** | | | **Down- CF** | | | **Thresholds** | |
|  | **z** | | **p** | **z** | | **p** | **Lower** | **Upper** |
| Cytoplasm | + | 5.67 | *** | + | 4.08 | *** | 416 | 672 |
| Nucleus | + | 6.10 | *** | + | 3.66 | *** | 462 | 743 |
| Unknown | - | 4.07 | *** | - | 1.86 | *** | 275 | 446 |
| Membrane | + | 4.95 | *** | + | 5.73 | *** | 458 | 742 |
| Mitochondrion | + | 5.77 | *** | + | 7.59 | *** | 436 | 719 |
| Endoplasmic reticulum | + | 2.29 | *** | + | 2.32 | *** | 384 | 597 |
| Endomembrane system | + | 2.91 | *** | + | 5.28 | *** | 468 | 775 |
| Mitochondrial envelope | + | 1.76 | *** | + | 3.74 | *** | 294 | 471 |
| Ribosome | + | 0.47 | 0.32 | - | 2.23 | *** | 214 | 324 |
| Plasma membrane | + | 3.07 | *** | + | 0.43 | 0.33 | 603 | 912 |
| Nucleolus | + | 4.13 | *** | + | 0.70 | 0.24 | 432 | 714 |
| Chromosome | + | 1.83 | *** | + | 2.44 | *** | 472 | 753 |
| Cytoskeleton | + | 1.57 | 0.06 | + | 0.26 | 0.40 | 549 | 896 |
| Vacuole | + | 2.14 | *** | + | 1.98 | *** | 476 | 762 |
| Golgi apparatus | + | 1.55 | 0.06 | + | 1.99 | *** | 474 | 765 |
| Site of polarized growth | + | 1.97 | *** | - | 0.19 | 0.42 | 655 | 995 |
| Bud | + | 2.12 | *** | - | 0.22 | 0.41 | 668 | 1025 |
| Cytoplasmic membrane-bound vesicle | + | 1.34 | 0.09 | + | 2.52 | *** | 500 | 832 |
| Cell cortex | + | 2.32 | *** | + | 0.71 | 0.24 | 665 | 1066 |
| Cell wall | + | 1.55 | 0.06 | - | 0.33 | 0.37 | 483 | 762 |
| Membrane fraction | + | 0.51 | 0.30 | + | 0.58 | 0.28 | 447 | 691 |
| Microtubule organizing center | + | 0.56 | 0.29 | + | 0.66 | 0.26 | 461 | 677 |
| Peroxisome | - | 0.25 | 0.40 | + | 0.22 | 0.41 | 407 | 577 |
| Extracellular region | + | 0.99 | 0.16 | + | 0.57 | 0.29 | 348 | 657 |
| Other | + | 0.65 | 0.26 | + | 0.39 | 0.35 | 401 | 590 |
|  |  |  |  |  |  |  |  |  |
