# Supplementary material for: Minimization of Biosynthetic Costs in Adaptive Gene Expression Responses of Yeast to Environmental Changes
Source: PLoS Comput Biol. 2010 Feb 12;6(2):e1000674. doi: 10.1371/journal.pcbi.1000674 (PMC2820516; doi:10.1371/journal.pcbi.1000674)
Supplement: Table S5 — Categorization by Function (Yeast Go-Slim): Molecular complexes and protein concentrations. For each group we computed the number and frequency of genes related to any molecular complex, and the mean and quartiles of protein concentrations. (0.06 MB DOC) [file pcbi.1000674.s009.doc]

| **Function** | **Complexes** | | **Protein Abundance** | | | |
| --- | --- | --- | --- | --- | --- | --- |
|  | **N** | **Freq** | **Mean** | **0.25** | **0.5** | **0.75** |
| Unknown | 137 | 0.05 | 4.31 | 0.72 | 1.71 | 3.42 |
| Hydrolase activity | 191 | 0.26 | 13.41 | 1.04 | 2.88 | 7.04 |
| Transferase activity | 125 | 0.18 | 9.65 | 1.04 | 2.48 | 6.57 |
| Protein binding | 139 | 0.27 | 13.54 | 0.77 | 2.59 | 6.65 |
| Transporter activity | 86 | 0.21 | 18.71 | 0.91 | 2.97 | 8.50 |
| Structural molecule activity | 280 | ***0.83*** | 30.17 | 1.82 | 6.22 | 31.59 |
| Transcription regulator activity | 134 | 0.41 | 3.05 | 0.54 | 1.36 | 3.51 |
| RNA binding | 155 | 0.48 | 10.55 | 1.32 | 2.88 | 7.79 |
| Oxidoreductase activity | 41 | 0.16 | 28.47 | 2.01 | 6.16 | 16.23 |
| DNA binding | 59 | 0.27 | 18.44 | 0.50 | 1.30 | 3.21 |
| Enzyme regulator activity | 43 | 0.24 | 8.11 | 0.66 | 1.77 | 5.98 |
| Peptidase activity | 40 | 0.26 | 11.80 | 0.87 | 4.72 | 11.43 |
| Protein kinase activity | 10 | 0.08 | 2.32 | 0.54 | 1.37 | 3.23 |
| Nucleotidyl transferase activity | 48 | 0.39 | 6.45 | 1.55 | 3.08 | 6.21 |
| Ligase activity | 24 | 0.20 | 19.20 | 0.70 | 2.89 | 17.77 |
| Helicase activity | 32 | 0.35 | 8.69 | 0.72 | 1.69 | 9.92 |
| Lyase activity | 6 | 0.07 | 40.61 | 2.04 | 5.41 | 13.61 |
| Signal transducer activity | 5 | 0.08 | 2.42 | 0.74 | 1.51 | 2.74 |
| Translation regulator activity | 40 | ***0.71*** | 40.23 | 1.55 | 10.03 | 34.48 |
| Isomerase activity | 2 | 0.04 | 19.44 | 2.14 | 4.40 | 9.57 |
| Phosphoprotein phosphatase activity | 12 | 0.27 | 5.05 | 0.71 | 3.97 | 7.22 |
| Motor activity | 10 | ***0.56*** | 1.34 | 0.22 | 0.96 | 2.18 |
| Other | 26 | 0.19 | 5.86 | 1.57 | 2.77 | 6.26 |
