# Supplementary material for: Minimization of Biosynthetic Costs in Adaptive Gene Expression Responses of Yeast to Environmental Changes
Source: PLoS Comput Biol. 2010 Feb 12;6(2):e1000674. doi: 10.1371/journal.pcbi.1000674 (PMC2820516; doi:10.1371/journal.pcbi.1000674)
Supplement: Table S6 — Categorization by Process (Yeast Go-Slim Molecular complexes and protein concentrations. For each group we computed the number and frequency of genes related to any molecular complex, and the mean and quartiles of protein concentrations. (0.07 MB DOC) [file pcbi.1000674.s010.doc]

| **Process** | **Complexes** | | **Protein Abundance** | | | |
| --- | --- | --- | --- | --- | --- | --- |
|  | **N** | **Freq** | **Mean** | **0.25** | **0.5** | **0.75** |
| Unknown | 14 | 0.01 | 3.11 | 0.59 | 1.44 | 3.26 |
| Organelle organization and biogenesis | 471 | 0.40 | 12.44 | 1.08 | 2.63 | 6.63 |
| Transport | 201 | 0.21 | 12.69 | 1.11 | 2.75 | 6.92 |
| Protein modification | 201 | 0.35 | 6.72 | 1.01 | 2.41 | 6.16 |
| Transcription | 246 | 0.49 | 4.28 | 0.77 | 1.73 | 4.49 |
| Protein biosynthesis | 313 | ***0.64*** | 30.86 | 1.88 | 6.08 | 30.07 |
| DNA metabolism | 223 | 0.46 | 10.78 | 0.75 | 1.75 | 4.08 |
| RNA metabolism | 218 | 0.46 | 7.31 | 1.43 | 2.80 | 6.49 |
| Response to stress | 89 | 0.21 | 15.28 | 0.78 | 2.44 | 6.40 |
| Cell cycle | 140 | 0.34 | 4.00 | 0.53 | 1.38 | 3.69 |
| Ribosome biogenesis and assembly | 139 | 0.44 | 14.49 | 2.60 | 5.22 | 12.88 |
| Vesicle-mediated transport | 36 | 0.12 | 7.70 | 1.08 | 2.50 | 6.27 |
| Morphogenesis | 30 | 0.12 | 5.42 | 0.69 | 1.63 | 4.28 |
| Generation of precursor metabolites and energy | 75 | 0.32 | 29.30 | 1.47 | 4.32 | 12.49 |
| Lipid metabolism | 12 | 0.05 | 9.77 | 0.78 | 2.19 | 6.70 |
| Cytoskeleton organization and biogenesis | 67 | 0.31 | 3.42 | 0.59 | 1.55 | 3.67 |
| Carbohydrate metabolism | 33 | 0.16 | 28.17 | 1.19 | 3.46 | 13.60 |
| Amino acid and derivative metabolism | 19 | 0.10 | 29.20 | 1.91 | 6.63 | 25.99 |
| Signal transduction | 13 | 0.07 | 5.68 | 0.72 | 1.52 | 3.95 |
| Protein catabolism | 76 | 0.47 | 13.76 | 1.36 | 3.20 | 9.97 |
| Cell wall organization and biogenesis | 13 | 0.08 | 7.34 | 0.74 | 1.93 | 4.38 |
| Meiosis | 28 | 0.20 | 3.07 | 0.43 | 1.40 | 3.34 |
| Cell homeostasis | 27 | 0.23 | 23.92 | 1.39 | 2.95 | 8.26 |
| Sporulation | 14 | 0.13 | 11.58 | 0.72 | 2.19 | 8.71 |
| Conjugation | 22 | 0.20 | 10.38 | 0.72 | 1.56 | 5.69 |
| Cytokinesis | 4 | 0.04 | 3.78 | 0.74 | 1.60 | 4.28 |
| Membrane organization and biogenesis | 13 | 0.14 | 5.17 | 0.74 | 1.89 | 4.44 |
| Cellular respiration | 30 | 0.36 | 7.52 | 1.36 | 3.39 | 7.92 |
| Cell budding | 10 | 0.13 | 3.99 | 0.64 | 1.47 | 4.29 |
| Vitamin metabolism | 0 | 0.00 | 8.97 | 0.62 | 3.06 | 5.63 |
| Pseudohyphal growth | 7 | 0.11 | 8.37 | 0.61 | 1.71 | 3.97 |
| nuclear organization and biogenesis | 28 | 0.56 | 13.13 | 1.42 | 3.01 | 8.00 |
| Electron transport | 23 | ***0.70*** | 10.75 | 2.96 | 5.49 | 10.24 |
| Other | 21 | 0.05 | 10.56 | 0.95 | 2.37 | 6.90 |
