# Supplementary material for: Minimization of Biosynthetic Costs in Adaptive Gene Expression Responses of Yeast to Environmental Changes
Source: PLoS Comput Biol. 2010 Feb 12;6(2):e1000674. doi: 10.1371/journal.pcbi.1000674 (PMC2820516; doi:10.1371/journal.pcbi.1000674)
Supplement: Table S7 — Categorization by Molecular Component(Yeast Go-Slim): Molecular complexes and protein concentrations. For each group we computed the number and frequency of genes related to any molecular complex, and the mean and quartiles of protein concentrations. (0.06 MB DOC) [file pcbi.1000674.s011.doc]

| **Component** | **Complexes** | | **Protein Abundance** | | | |
| --- | --- | --- | --- | --- | --- | --- |
|  | **N** | **Freq** | **Mean** | **0.25** | **0.5** | **0.75** |
| Cytoplasm | 670 | 0.20 | 14.04 | 1.08 | 2.73 | 7.39 |
| Nucleus | 664 | 0.35 | 7.94 | 0.91 | 2.25 | 5.41 |
| Unknown | 2 | 0.00 | ***1.94*** | ***0.34*** | ***0.81*** | ***1.80*** |
| Membrane | 207 | 0.19 | 11.51 | 0.94 | 2.58 | 6.35 |
| Mitochondrion | 242 | 0.24 | 10.31 | 1.08 | 2.54 | 6.86 |
| Endoplasmic reticulum | 30 | 0.09 | 10.54 | 1.21 | 2.84 | 6.76 |
| Endomembrane system | 97 | 0.34 | 8.21 | 1.14 | 2.86 | 7.38 |
| Mitochondrial envelope | 87 | 0.31 | 11.20 | 1.21 | 3.23 | 6.86 |
| Ribosome | 265 | ***0.99*** | 45.72 | 3.09 | 13.93 | 49.25 |
| Plasma membrane | 15 | 0.06 | 19.82 | 0.67 | 1.68 | 5.37 |
| Nucleolus | 110 | 0.49 | 9.81 | 2.02 | 4.28 | 9.60 |
| Chromosome | 99 | 0.45 | 16.86 | 0.80 | 1.73 | 4.05 |
| Cytoskeleton | 53 | 0.26 | 3.33 | 0.63 | 1.53 | 3.46 |
| Vacuole | 17 | 0.09 | 12.23 | 0.68 | 2.01 | 4.44 |
| Golgi apparatus | 28 | 0.16 | 8.66 | 1.62 | 3.09 | 7.66 |
| Site of polarized growth | 8 | 0.05 | 2.35 | 0.45 | 1.13 | 2.68 |
| Bud | 9 | 0.06 | 2.35 | 0.47 | 1.23 | 2.61 |
| Cytoplasmic Membrane-bound vesicle | 16 | 0.16 | 10.20 | 1.58 | 3.13 | 9.54 |
| Cell cortex | 6 | 0.06 | 6.84 | 0.67 | 1.63 | 4.91 |
| Cell wall | 3 | 0.03 | 34.25 | 1.43 | 6.27 | 23.64 |
| Membrane fraction | 6 | 0.07 | 6.52 | 0.68 | 2.22 | 5.77 |
| microtubule Organizing center | 13 | 0.22 | 2.69 | 0.32 | 0.96 | 3.33 |
| Peroxisome | 1 | 0.02 | 2.65 | 0.59 | 1.24 | 2.57 |
| Extracellular region | 0 | 0.00 | 5.87 | 0.54 | 1.46 | 7.89 |
| Other | 65 | ***0.51*** | 7.52 | 0.78 | 3.92 | 8.73 |
